# The 3D dynamic visualization simulation of rice plant based on morphological structure model and the application in phenotypic calculation

**DOI:** 10.1371/journal.pone.0309052

**Published:** 2024-11-21

**Authors:** Yonghui Zhang, Yujie Zhang, Peng Zhang, Liang Tang, Xiaojun Liu, Weixing Cao, Yan Zhu

**Affiliations:** 1 School of Computer Engineering, Weifang University, Weifang, P. R. China; 2 Weifang People’s Hospital, Weifang, P. R. China; 3 School of Physics and Electronic Information, Weifang University, Weifang, P. R. China; 4 National Engineering and Technology Center of Information Agriculture, Nanjing Agricultural University, Nanjing, P. R. China; Shandong Agricultural University, CHINA

## Abstract

The virtual crop stands as a vital content in crop model research field, and has become an indispensable tool for exploring crop phenotypes. The focal objective of this undertaking is to realize three-dimensional (3D) dynamic visualization simulations of rice individual and rice populations, as well as to predict rice phenotype using virtual rice. Leveraging our laboratory’s existing research findings, we have realized 3D dynamic visualizations of rice individual and populations across various growth degree days (GDD) by integrating the synchronization relationship between the above-ground parts and the root system in rice plant. The resulting visualization effects are realistic with better predictive capability for rice morphological changes. We conducted a field experiment in Anhui Province in 2019, and obtained leaf area index data for two distinct rice cultivars at the tiller stage, jointing stage, and flowering stage. A method of segmenting leaf based on the virtual rice model is employed to predict the leaf area index. A comparative analysis between the measured and simulated leaf area index yielded relative errors spanning from 7.58% to 12.69%. Additionally, the root mean square error, the mean absolute error, and the coefficient of determination were calculated as 0.56, 0.55, and 0.86, respectively. All the evaluation criteria indicate a commendable level of accuracy. These advancements provide both technical and modeling support for the development of virtual crops and the prediction of crop phenotypes.

## 1 Introduction

Virtual crops have emerged as a pivotal topic in intelligent agriculture, boasting immense potential for application in agricultural education, plant-type design, and crop phenotype analysis [1, 2]. Significant advancements have been achieved in the morphological modeling and visualization of various crops, including maize [3–6], wheat [7–10], cotton [11–13], and crop root systems [14, 15]. In addition, there have been remarkable breakthroughs in the development of crop functional-structural models [16, 17]. These advancements not only enhance our understanding of crop growth and development but also pave the way for more precise and efficient agricultural practices. Rice crop, a vital food crop with its intricate morphological structure, has consistently garnered significant attention as a research hotspot in the virtual crop. In recent years, Watanabe et al. [18] visualized a single rice plant utilizing L-studio, leveraging a rice organs morphology model that demonstrated a remarkable prediction accuracy in rice tillering. Zheng et al. [19] constructed a 3D model of the rice canopy using a virtual layer cutting method. This method was grounded in 3D structural data obtained from a field-based 3D digitizing instrument. Furthermore, the 3D digitizing data were utilized to simulate and compare light distribution within rice canopies of different varieties, thereby investigating their photosynthetic production potential [20]. Ding et al. [21] employed a parameterized L-system to generate the topological structure and achieved 3D visualization of rice plant through the integration of organ geometry modeling. Xu et al. [22] established a gene-related functional structure model of rice to improve the research depth and breadth. Subsequently, Xu et al. [2] put forward the problems and disadvantages of using rice growth model for virtual seed breeding and discussed the possible solutions. Wei et al. [23] conducted a quantitative analysis to assess the impact of the PAY1 gene on the structural characteristics of rice canopy, aiming to provide valuable structural parameters for breeding the desired plant type. Furthermore, our laboratory has reported several meaningful results in modeling morphology of rice plant. Chang [24] established a morphology model of the above-ground parts in rice plant, drawing from field experimental data under different growth conditions. Building on this foundation, Wu [25] achieved visualization expressions for rice organs, plant individual, and rice populations without root system. Zhu et al. [26] further constructed a dynamic leaf shape model for various rice cultivars across different growth environments. Meanwhile, we have developed models for rice leaf morphology [27], stem-sheath angle [28], panicle morphology and panicle color [29], and leaf color [30], all aimed at improving the morphological modeling and visualization for rice plant. These achievements provide crucial insights for crop growth prediction, cultivation management, and plant type design [31]. Nevertheless, there is a lack of systematic studies on the visualization of rice plant that integrate both the above-ground parts and root system, as well as population-level.

Crop phenotype is played a crucial role for photosynthesis during crop growth, which can be studied using conditional method [32], but it is insufficient and destructive. Recently, some non-destructive monitoring methods have been applied to obtain and predict the crop phenotype traits based on sensor techniques [33–38]. The segmentation technique for crop organ based on 3D point clouds also had attracted much attention, providing refined data for phenotype extraction and growth simulation [39–41]. However, the intricate structure of the crop canopy and the challenges associated with obtaining accurate morphological data have hindered the simulation on crop phenotype. The virtual crop model holds the potential to offer real-time and dynamic simulations of crop phenotypes during various growth stages, under different crop growth conditions, but it has been infrequently employed for predicting canopy phenotype traits.

**Fig 1:**
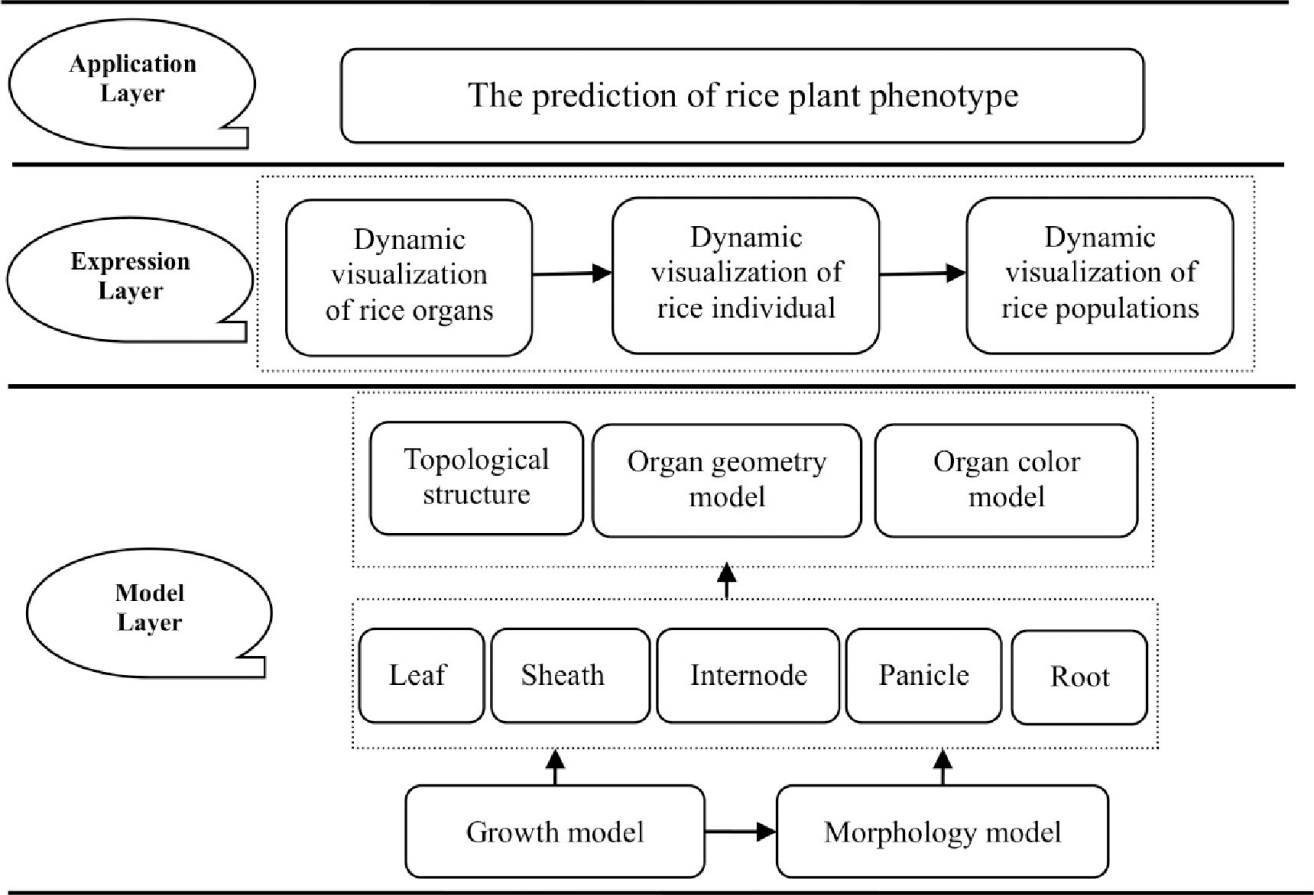
Basic research framework in this study.

According to the research framework described in Fig 1, the objectives of this study are to (1) realize the 3D dynamic visualizations of rice individual and populations through a systematic integration of our lab’s previous studies [24, 26–30] using the computer visualization techniques. (2) to employ the virtual rice model developed in this study to simulate the plant phenotype of leaf area index (LAI) for diverse rice cultivars across various growth conditions and stages. These simulations will offer invaluable support for the construction of virtual crop models and their subsequent application in crop production and management strategies.

## 2 Materials and methods

### 2.1 Field experiment design

The field experiment was conducted in 2019 in Dangtu of Anhui Province (31°34’15″N, 118°29’52″E). Two cultivars, Wuxiangjing 14 (WJ14) and Yangdao 6 (YD6), were planted on 20 May. The transplantation occurred on 7 June, with YD6 planted at a spacing of 26 cm × 18 cm and WJ14 at 18 cm × 15 cm, each hole containing one seedling per cultivar. The experimental design was a randomized complete block with three replications. For both cultivars, totaling nitrogen rate (230 kg·ha^-1^) was applied in four splits, 50% pre-transplanting, 10% at tillering stage, 20% at spikelet promotion stage, and 20% at spikelet protection stages. Phosphorus (P_2_O_5_) and potassium (K_2_O) were applied as basal doses at 80 kg·ha^-1^ and 160 kg·ha^-1^, respectively. All other management measures adhered to local cultural practices to optimize potential productivity.

### 2.2 Data acquisition

For each replication, three rice plants from each cultivar were destructively sampled at the tillering stage, jointing stage, and flowering stage. Utilizing the LI-3100 (LI-COR, LI-3000C, USA) leaf area instrument, the leaf area of each leaf was measured for all plant samples. Subsequently, the average LAI of each cultivar was computed, considering the planting density. Furthermore, daily meteorological data were obtained from the meteorological information center of the State Meteorological Administration of China.

### 2.3 Spatial topology structure of rice plant

**Fig 2:**
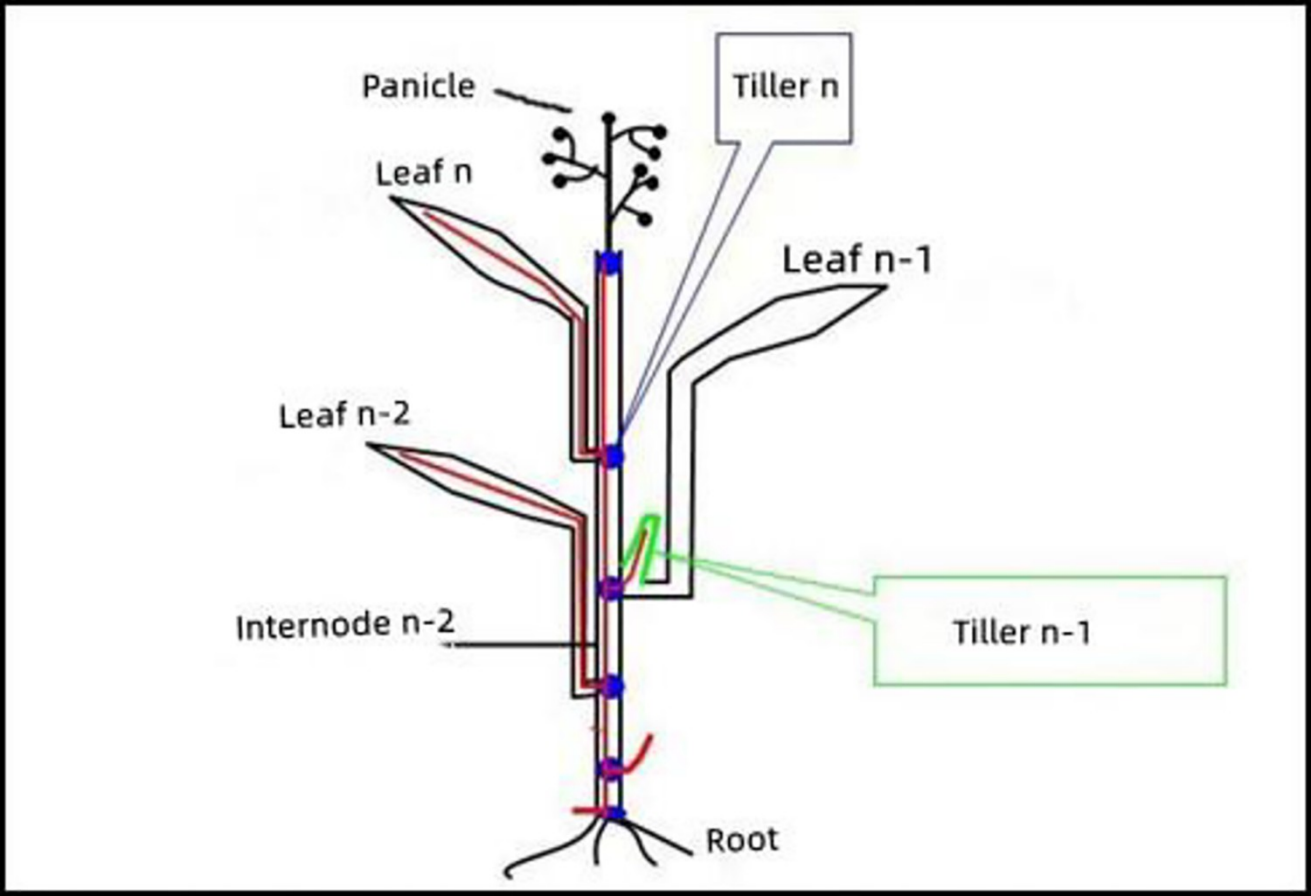
Basic structure of main stem in rice plant.

The rice plant comprises a main stem and several tillers. The main stem is made up of nodes and internodes, the leaves are opposite and grow on nodes through leaf sheaths. The panicle, on the other hand, emerges from the panicle neck node. If the nodes, internodes, leaves, and sheaths that grow on a single node are considered as a leaf growth unit, the main stem can be divided into several structurally similar but differently sized units, including the leaf growth unit, panicle unit, and root unit (as depicted in Fig 2). The tillers share a similar structure with the main stem, but they form a specific angle with the main stem.

### 2.4 Synchronous relationships of organs among above-ground parts of rice plant

According to the research findings [42], when the *n*^th^ leaf emerges on the main stem, the *n*^th^ leaf sheath and the (*n+*1)^th^ leaf undergo elongation, while the internodes between the (*n-*1)^th^ and (*n-*2)^th^ leaves also elongate. At jointing stage, the lower leaf internodes generally remain stationary, and begin to elongate after jointing stage. For the rice cultivar with *LN* leaves and *m* elongated internodes on main stem, the time of jointing stage accurately determined using the equation:

physiologicaljointingstage=m‐2reciprocalleafage=LN‐m+3leafage (1)

When the *n*^th^ leaf on the main stem starts to appear, the first leaf on tillers at (*n-*3)^th^ leaf position and the second leaf on tillers at (*n*-4)^th^ leaf position also appear at the same time [43].

When the uppermost internode on the stem starts to elongate, the panicle emerges from the leaf sheath of the flag leaf, a process known as heading. Heading spans approximately 5 days, commencing with the emergence of the top panicle and culminating with the emergence of the uppermost internode. Subsequently, it takes around 7 to 9 days for the uppermost internode to reach its fixed length, an interval that roughly corresponds to the duration of a leaf cycle. Therefore, the emergence, fixed length time of each leaf, leaf sheath and rice panicle at different leaf positions on the main stem or tiller, as well as the elongation and fixed length time of each internode can be calculated by the method of Jiang et al. [43].

### 2.5 Synchronous relationships between above-ground part and root system

Previous research conducted by Chang et al. [44], and Hu and Ding [42], coupled with experimental observations, reveal that the timing of root emergence in rice plant follows specific patterns. Specifically, adventitious roots first appear on the coleoptile segment upon the emergence of the first leaf on the main stem. Subsequently, as the second leaf emerges, approximately five adventitious roots grow out on the coleoptile segment. Then, with the emergence of the third leaf, roots on the incomplete leaf segment become visible. Notably, when the (*n*+3)^th^ leaf emerges on the main stem, both the (*n*+2)^th^ leaf sheath and the root on the *n*^th^ leaf segment commence elongation. These observations indicate the existence of a precise synchronicity between the roots and leaves of the main stem. Therefore, the initial elongation time of adventitious roots on the *i*^th^ root segment can be accurately determined by referencing the emergence time of the corresponding leaves on the main stem, as outlined by Jiang et al. [43].

The growth of root branch is late for one leaf cycle behind root [45], when the *n*^th^ leaf emergence, the (*n-*3)^th^ leaf segment root begins to grow out, (*n*-4)^th^ leaf segment root is branched once, and the (*n*-5*)*^th^ leaf segment root is branched twice. Therefore, the initial occurrence time of branching roots at different levels can be also calculated [43]. The number of root nodes of the main stem and tiller stems of rice plant can be obtained according to the total number of leaves on the main stem and the number of elongation internodes in a certain rice variety [46].

### 2.6 The application of virtual rice on plant phenotype

LAI serves as a crucial indicator of rice phenotype in variety breeding and yield prediction. Through real-time monitoring and predicting of rice LAI, we can know the growth of rice and provide scientific basis for rice production and management. In this study, we use a method of segmenting leaf based on the virtual rice model to forecast the LAI for two rice cultivars at different growth stages, the steps are summarized as follows:

**Step 1:** Through our previous studies [26], we established a method to determine the axis length and leaf width of any point on the leaf veins for each rice leaf, described by Eqs (2) and (3), respectively.


$$LLn(GDD)=\frac{LLn}{1+La\times e^{\frac{-Lb\times (GDD-{IGDD}_{n})}{{\Delta GDD}_{n}}}}\times min(FN,FW)$$ (2)

Where, *LL*n (GDD) represents the length of leaf *n* on the main stem at a specific GDD time. *LL*n denotes the final length of leaf *n*. *IGDD*n is the initial GDD of leaf *n*.

△*GDD*n is the cumulative GDD required for the development and growth of leaf *n*. *FN* and *FW* are nitrogen factor and water factor, respectively. The detailed computations of the aforementioned parameters are outlined in prior research [26], which also delves into the length dynamics of leaves found on tillers. The coefficients La and Lb are assigned values of 8.65 and 6.26, respectively.


$$LWidn(LLen)=\left\{ \begin{array}{c} P\times {LLen}^{WLR},\;n=1,\;LN;\\ WPd\times {LLen}^{2}+WPe\times LLen,\;2\leq n\leq LN-1. \end{array}\right.$$ (3)

Here, *LWid*n (*LLen*) denotes the leaf width of leaf *n*, where the leaf length is *LLen*. *LN*, serving as a cultivar parameter, represents the ultimate count of leaves present on main stem. *P*, *WLR*, *WPd*, and *WPe* are equation coefficients. These parameters and coefficients were calculated by Zhu et al. [26].

**Fig 3:**
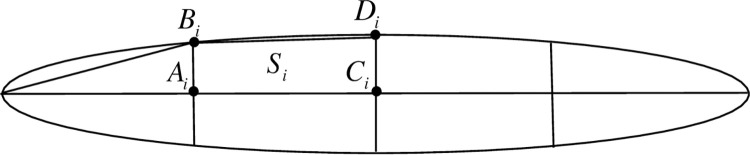
Structure diagram of rice leaf segmentation.


As depicted in Fig 3, each leaf can be segmented into *sn* segmentations with *sn*-1 line segments perpendicular to the leaf vein, each segmentation is composed of two symmetrical trapeziums. The area of each trapezium can be calculated using a specific Eq (4), and the large *sn* can allow us to quantify the overall leaf area with precision according to the principles of calculus.

$$S_{i}=\frac{\left(|A_{i}B_{i}|+|C_{i}D_{i}|)\times |A_{i}C_{i}\right|}{2}$$ (4)

Thus, the leaf area can be calculated by Eq (5),

$$S=2\times \sum_{i=1}^{N_{sp}}S_{i}$$ (5)

Where, *S* is the area of a rice leaf, *S*_i_ is the area of the *i*^th^ segmentation in each half leaf. *N*_*sp*_ is the number of segmentations in each leaf (Fig 3).

**Step 2:** Geometry morphology and leaf area of each leaf on rice tillers can be calculated according to the synchronous relationships [47]. Tillers number is simulated by the Eqs (6) and (7), which is a dynamic model of tiller number [48], then we can calculate the total area for all leaves in the single plant.


$$APPSTN_{i}=APPST\;N_{i-1}+\Delta APPSTN_{i}$$ (6)

$$\Delta APPSTN_{i}=T_{v}\times \Delta PPSTN_{i}\times FL\times min(NF,\;WDF)$$ (7)

Where APPSTN_i_ and ΔAPPSTN_i_ are the actual number and the actual increase of tillers for rice plant on the *i*^th^ day after rice emergence, respectively. T_v_ is the coefficient of tillering power for rice variety. FL, NF and WDF are different influence factors. These coefficient and factors were calculated by Meng [48].

**Step 3:** The total leaf area can be obtained by the step 1 and step 2, then we can calculate LAI (total leaf area/land area) with GDD according to the planting density.

### 2.7 The evaluation criteria

The differences between the simulated and measured values are assessed using the relative error (RE), calculated via Eq (8), along with the root mean square error (RMSE), mean absolute error (MAE), and the coefficient of determination (R^2^), as outlined by Zhang et al. [49].

$$RE=\frac{100\times |O_{i}-S_{i}|}{O_{i}}\%$$ (8)

where *O*_i_ and *S*_i_ are the observed value and the simulated value, respectively.

## 3 Results

The visualizations of both above-ground parts and root system in rice plant are primarily achieved through synchronization and topological relationships among the organs of each respective system. The visualization of rice individual is intricately linked with the synchronization relationship between the above-ground part and the root system. Moreover, rice populations are primarily comprised of individual plants that exhibit certain variations. Consequently, the visualization of plant populations goes beyond merely replicating individual plants, it necessitates the expression of these individual differences. This can be effectively realized through the employment of differentiated approaches.

### 3.1 The visualization of above-ground part of rice plant

The main stem of rice is divided into several growth units consisting of nodes, internodes and leaves, and rice panicle. Our simulations have captured the changes in geometric morphology of above-ground organs on main stem, as they occur with increasing GDD [24, 26]. Additionally, we have simulated spatial morphology features of leaf curves, panicle curves, and the angle between leaf and sheath on main stem [27–29]. The rice panicle, with its intricate structure, comprises primary branches, secondary branches, spikelets, and the panicle axis. Zhang et al. [29] provided a detailed description of its morphological construction and 3D dynamic visualization.

The number of growth units on tillers can be determined by leveraging the synchronization relationship between tillers and the main stem. The geometric morphology of organs within the growth unit of tillers at various GDD can be derived using the quantitative relationship established between tillers and the main stem [47]. Additionally, the spatial morphological parameters and color rendering are simulated based on the models employed for the main stem. The angle formed between the tiller and the main stem can be simulated using the method proposed by Watanabe et al. [18]. It’s worth noting that tiller number is influenced by the variety and growing environment, which can be predicted using a rice growth model [48].

**Fig 4:**
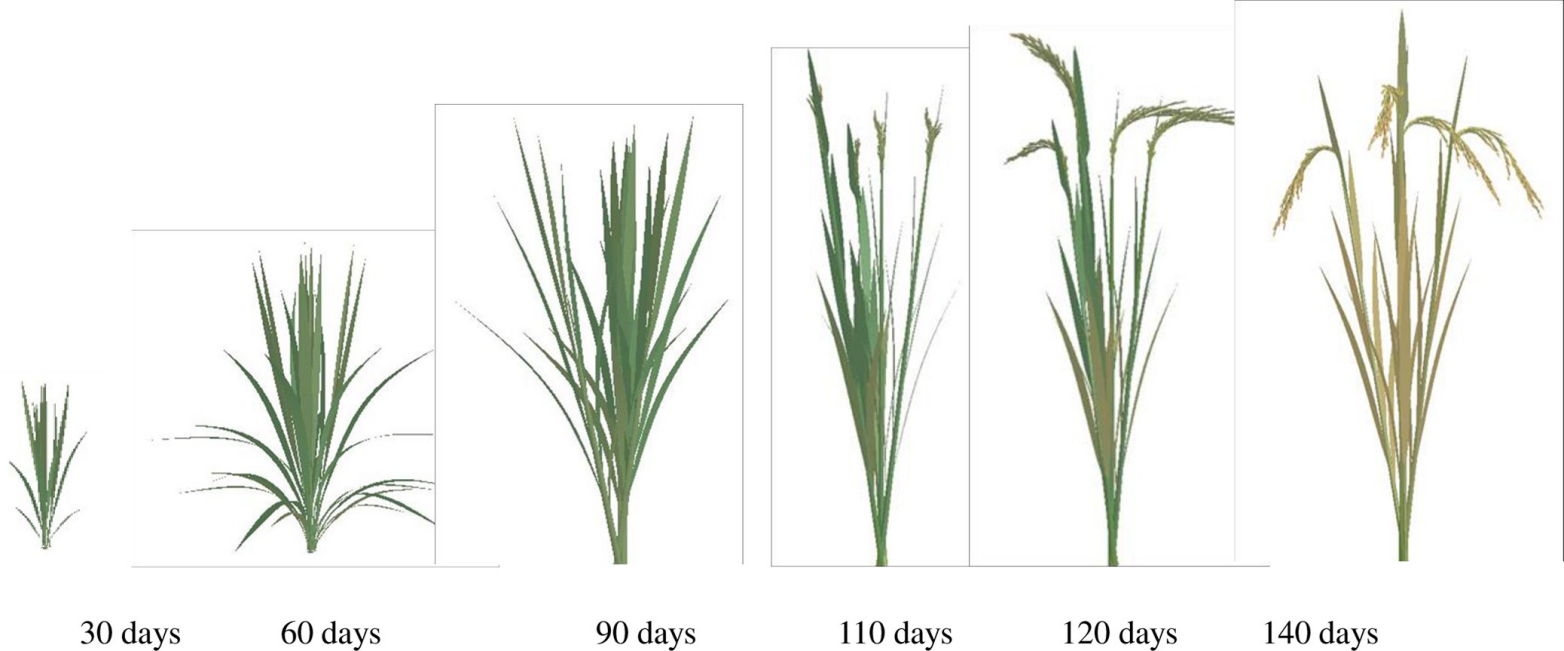
3D visualization of above-ground part in YD6 under low nitrogen level at different growth days.

**Fig 5:**
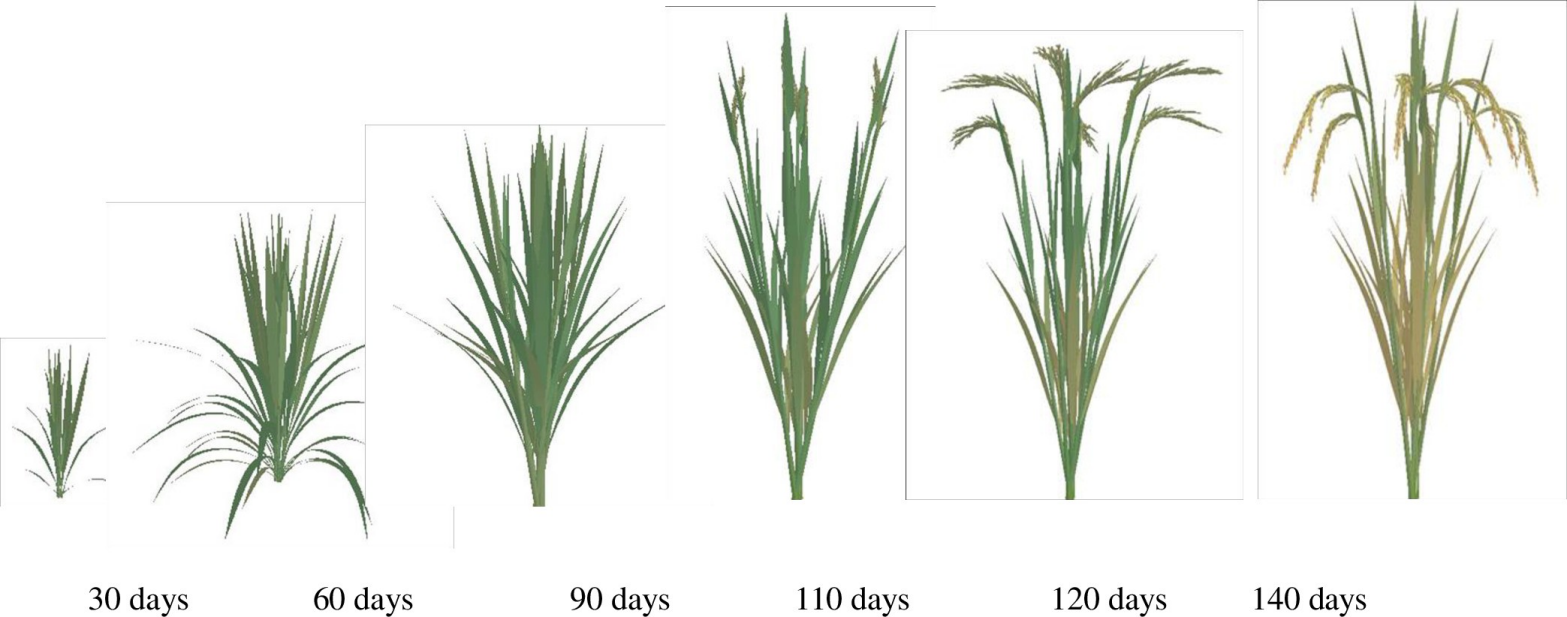
3D visualization of above-ground part in YD6 under normal nitrogen level at different growth days.

**Fig 6:**
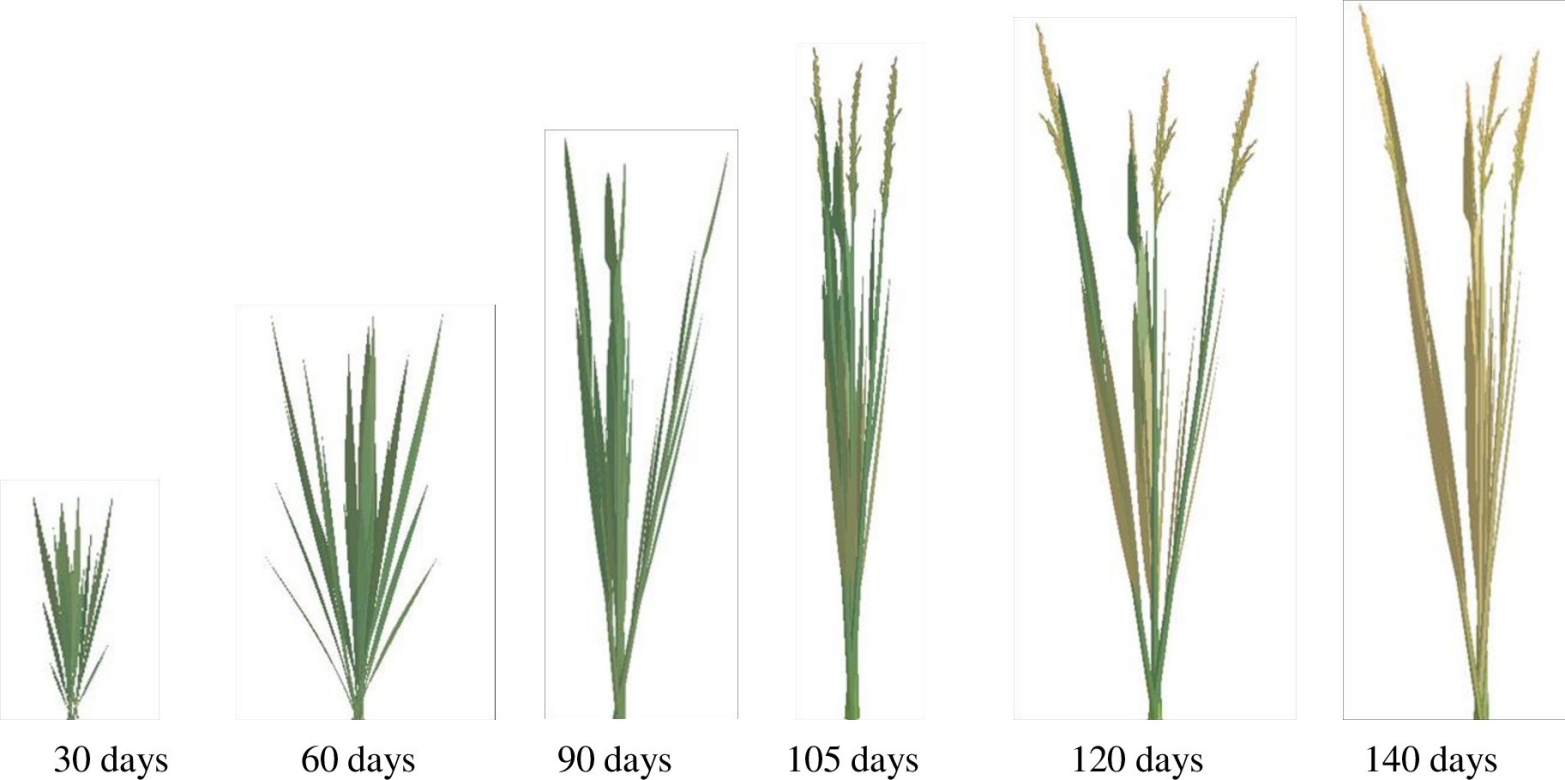
3D visualizations of above-ground part in W14 under low nitrogen level at different growth days.

**Fig 7:**
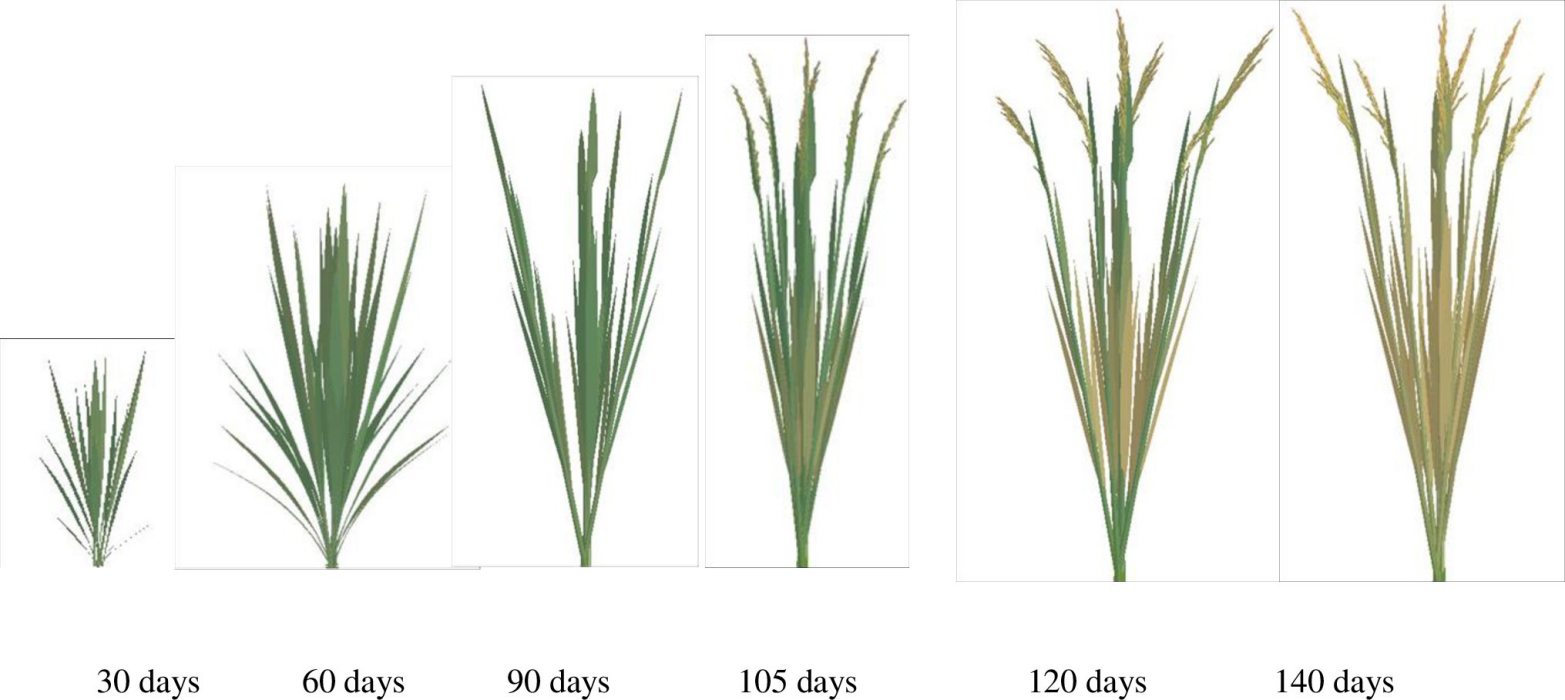
3D visualizations of above-ground part in W14 under normal nitrogen level at different growth days.

Based on our lab’s extensive research results, including morphology models, organ geometry models [24, 26], such as leaf length and width, leaf sheath length and diameter, and internode length and diameter, leaf curve models [27], stem-sheath angle models [28], leaf color models [30], and panicle color and morphology models [29], we have realized 3D dynamic visualization simulations of the above-ground parts of rice varieties YD6 and W14. These simulations capture the morphological changes in the rice plants over GDD time, under various growth conditions. The simulations depicted in Figs 4–7 were achieved using C#. NET and OpenGL techniques, adhering to the intricate rules of rice growth and the topological structure of its above-ground components.

The visualization results effectively capture the variations in morphological indices among rice plants grown under different varieties and nitrogen treatments. Notably, there are obvious morphological differences between the two plant types of W14 and YD6, as well as among crops of the same variety grown under different nitrogen application levels (Figs 4–7). These results can provide a comprehensive and intuitive understanding of the impact of these factors on rice growth.

### 3.2 The visualizations of root system

**Fig 8:**
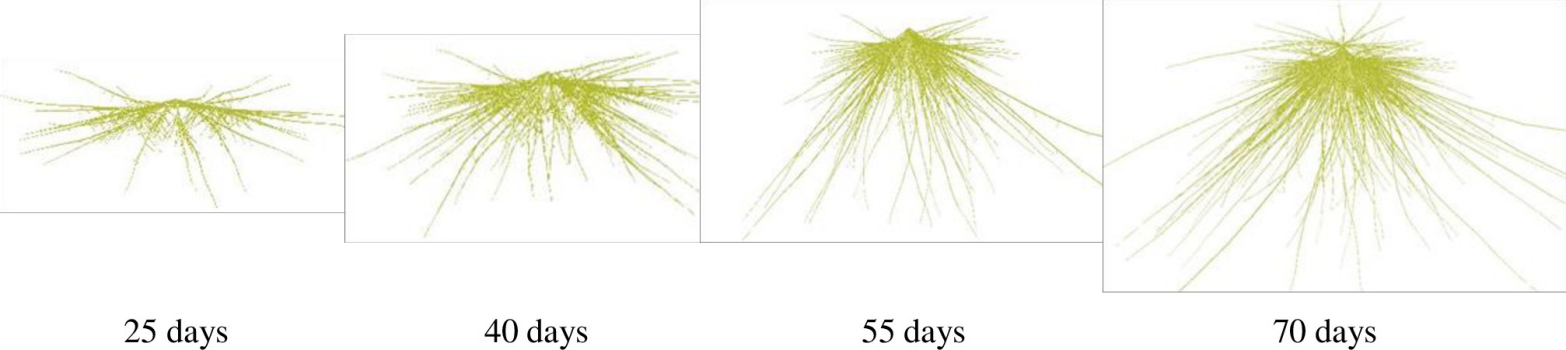
3D visualizations of root system in YD6 at different days after sowing under normal water and nitrogen conditions in rice plant.

**Fig 9:**
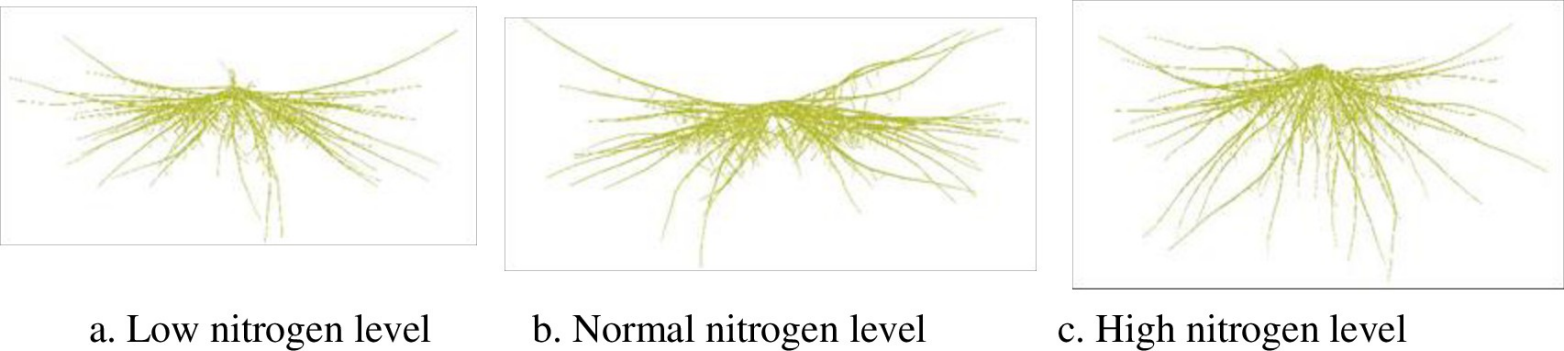
3D visualizations of root system in W14 under normal water and different nitrogen conditions at 40 days after sowing in rice plant.

The rice root system exhibits a whisker structure, encompassing both seed roots and adventitious roots. The seed root, originating from the radicle, is unique in its emergence. As the leaves develop, adventitious roots gradually differentiate, emerging from the base upwards along the root nodes. Primary branching roots sprout from these adventitious roots, while secondary branching roots arise from the primary ones. Notably, under conditions favoring high yields, rice roots exhibit a greater propensity for branching, as observed by Xu et al. [14]. Drawing upon the previous studies and experimental data pertaining to rice root morphology of our lab [14, 50, 51], 3D dynamic simulation of rice roots is achieved combined with the topological structure of rice roots through graphics rendering technique. The results demonstrate excellent dynamic predictions of the rice root system (Figs 8, 9).

### 3.3 The visualization of the whole rice plant

**Fig 10:**
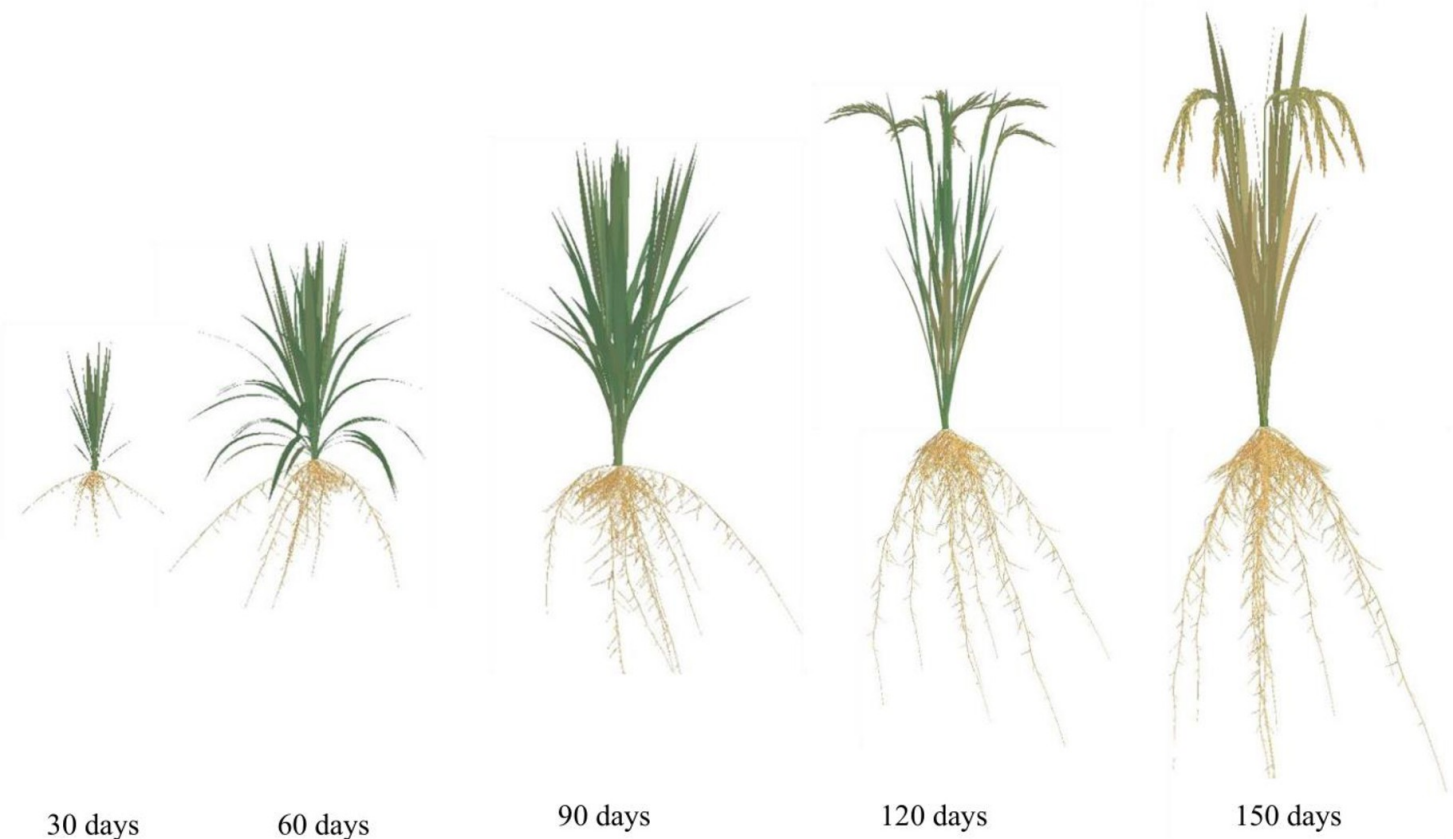
3D visualizations of the whole rice plant in YD6 at different growth stages under normal nitrogen level.

Rice plant comprises both an above-ground part and a root system. Leveraging the visualization results for both components, and a synchronization relationship between them, we achieve 3D dynamic visualizations of the entire rice plant over GDD using programming techniques (Fig 10). The simulation outcomes are highly realistic, and the simulation processes closely adhere to the laws governing the growth and development of rice plants. This includes the sequential appearance, expansion, maintenance, and eventual demise of rice organs. These findings demonstrate that our method effectively predicts changes in the geometric shape, spatial morphology, and spatial topological structure of rice plants throughout their lifecycle.

### 3.4 The visualization of rice plant populations

Rice populations are comprised of diverse plant individuals, each exhibiting unique structural and morphological differences. As such, the visualization of rice populations goes beyond mere replication of individual plants, it aims to comprehensively capture these individual differences. The distinctions between rice plants of the same or different varieties grown under identical or varying conditions, are primarily manifested in structural and morphological parameters, which can be extracted from the rice growth model [48] and morphology model [24, 26–29], respectively. Additionally, even within the same variety and growth conditions, the size (or number) of identical organs and the angles between organs at the same location can vary randomly within a specified range [*M-d*, *M+d*], where *M* represents the average size (or number) of the organs, and *d* signifies the standard deviation. To further enhance the diversity among rice individuals, we employ random rotation angles for plant organs and individuals, as well as random allocation of tiller numbers.

To improve the visualization efficiency, we utilize a grid model simplification technique that effectively reduces the number of faces, edges, and vertices of the model while maintaining its original geometric characteristics [52]. This approach is applied based on the distance between rice individuals and the viewpoint within the drawing scene. Furthermore, we employ LOD (Level of Detail) models with varying resolutions for plant individuals situated at different distances from the viewpoint, thereby optimizing the rendering efficiency of large populations.

**Fig 11:**
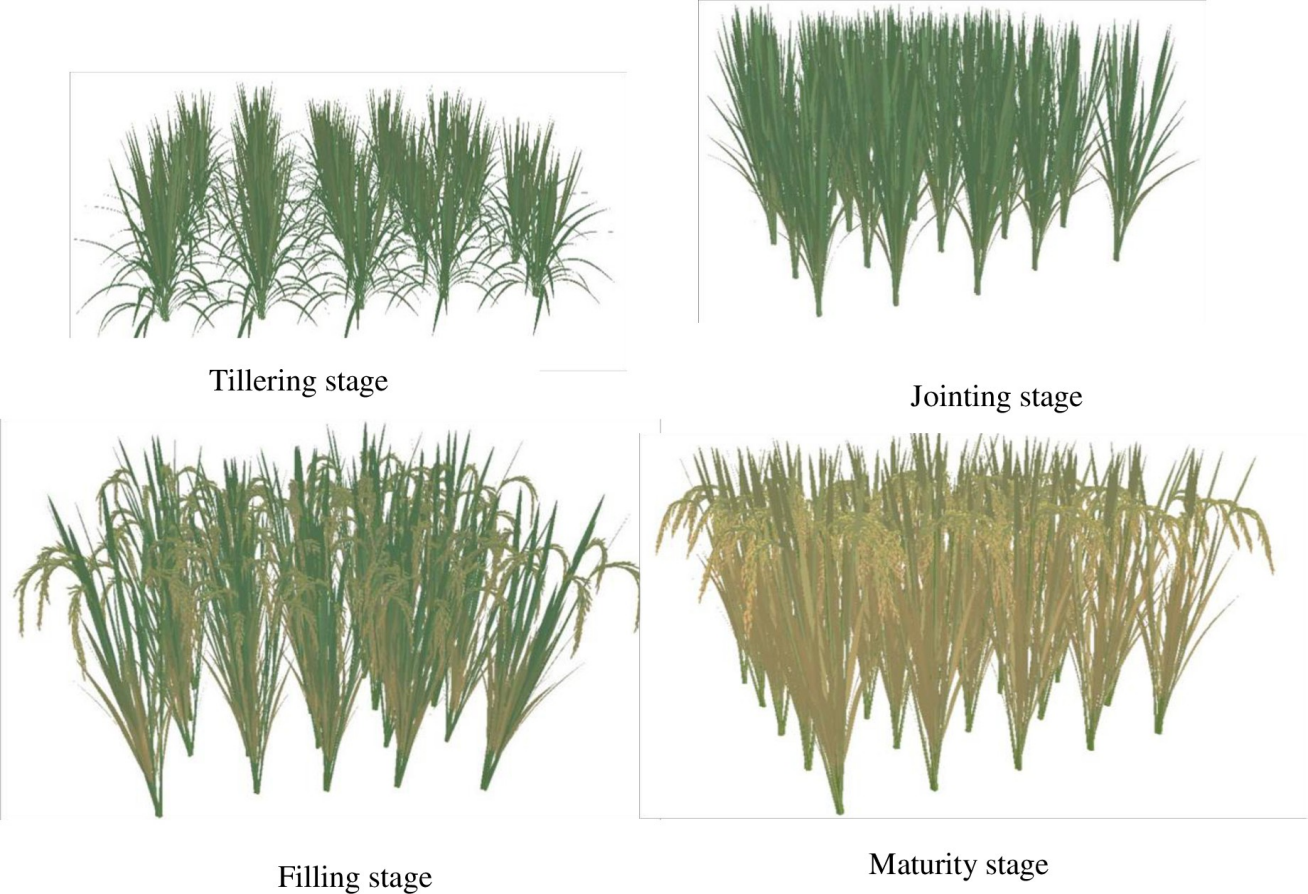
3D visualizations of plant population of YD6 at different growth stages.

**Fig 12:**
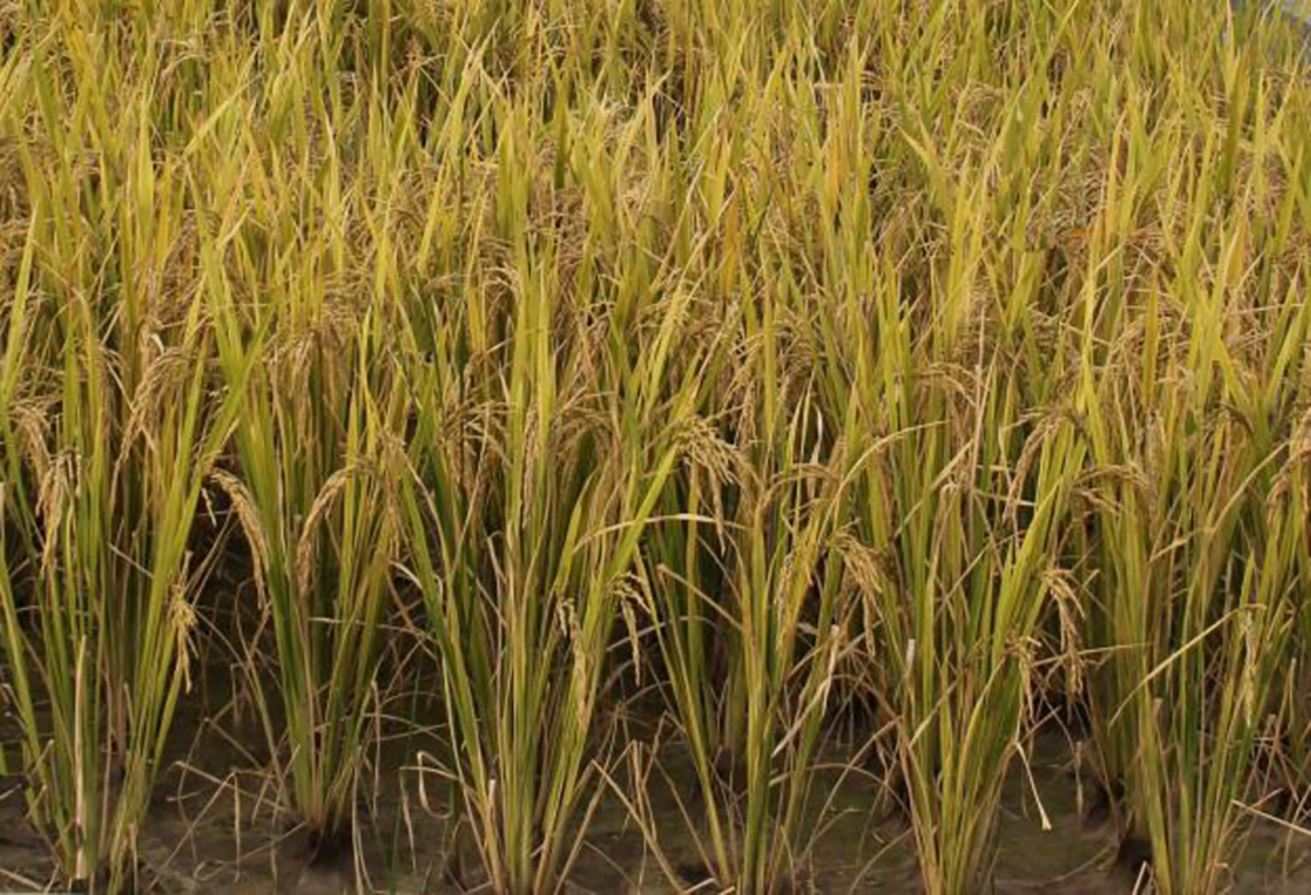
Real pictures of population in YD6 at maturity stage.

In scenarios involving a large population of rice plants, the display list technology can be employed to replicate a group of individuals exhibiting variations according to the situation of individual scale. This allows for the entire group to be randomly rotated and scaled in or out, creating a distinct difference from the original set of rice individuals. Utilizing the above rules and visualization techniques, and considering the variety of YD6 as a case under normal growth conditions, we have conducted 3D dynamic visualizations of rice populations (consisting of 5×5 plants) with row and column spacing of 30cm×30cm throughout various growth periods (Fig 11). The simulation effects are quite impressive when compared to real images of rice populations (Fig 12).

### 3.5 The simulation for the LAI of rice plant

**Fig 13:**
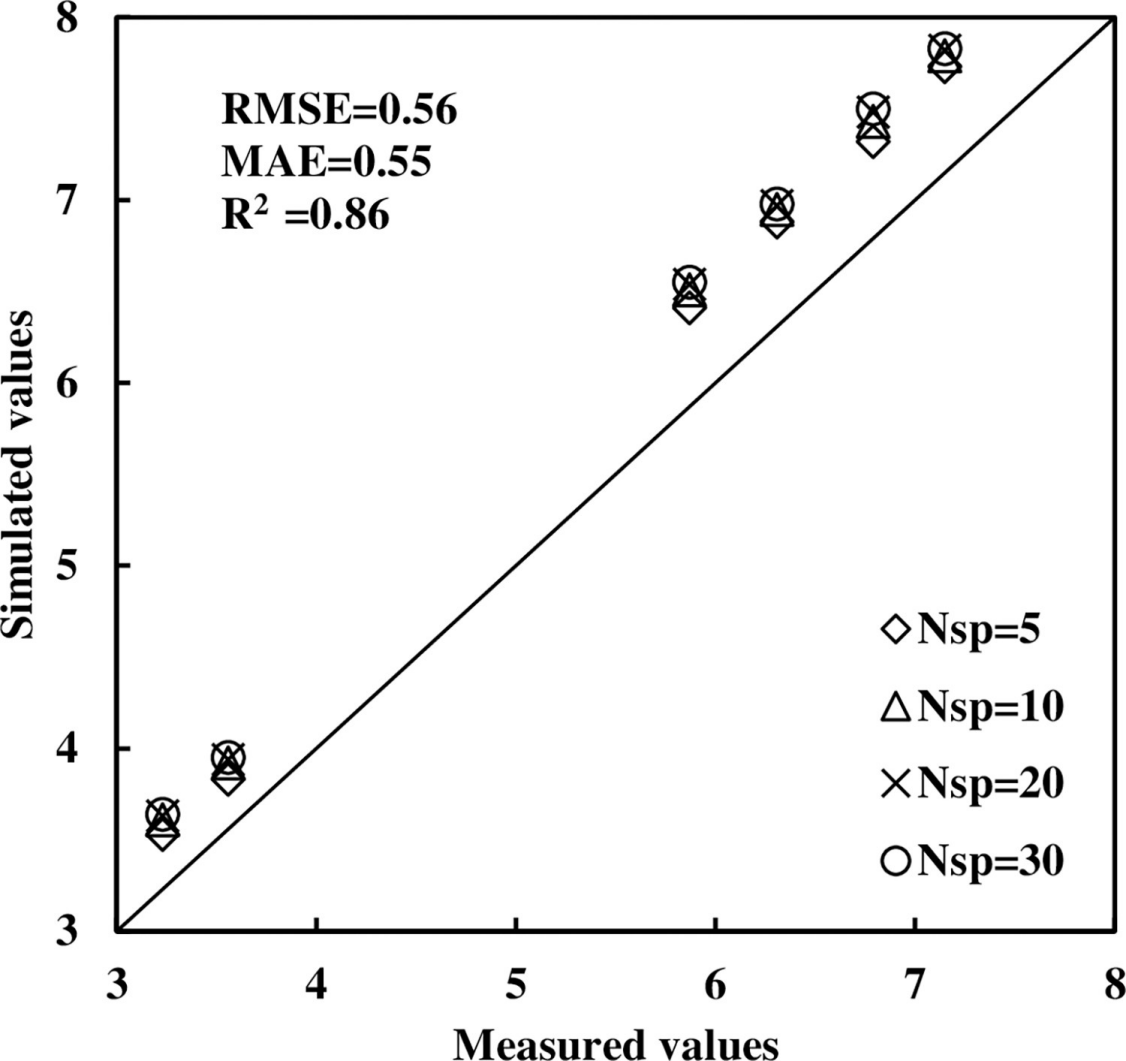
Comparisons between all measured and simulated LAI. *N*_sp_ is the number of splits of each leaf (Fig 3).

For two rice cultivars, the average measured LAI was recorded at 32 days, 50 days, and 65 days after transplanting, corresponding to the tillering stage, jointing stage, and flowering stage, respectively. Our virtual rice model was then utilized to simulate the LAI for two cultivars at the corresponding growth stages. Comparisons between the measured and simulated LAI, REs ranging from 7.58% to 12.69%, indicating a satisfactory level of accuracy across different segmented granularities of the leaves (Table 1). Furthermore, upon a thorough comparison of all the measured and simulated data, we have determined that the RMSE stands at 0.56, the MAE at 0.55, and the R^2^ value is 0.86. These results demonstrate the superior simulation effectiveness of our virtual rice model for predicting rice LAI (Fig 13).

**Table 1:** Comparisons between measured and simulated LAI at different growth stages under different segmentation granularity of rice leaf.

Cultivar	Growth stage	MLAI	SLAI(*N*_sp_ = 5)	RE	SLAI (*N*_sp_ = 10)	RE	SLAI (*N*_sp_ = 20)	RE	SLAI (*N*_sp_ = 30)	RE
YD6	Tiller stage	3.56	3.83	7.58	3.92	10.11	3.94	10.67	3.95	10.96
	Jointing stage	6.31	6.88	9.09	6.95	10.14	6.97	10.46	6.98	10.62
	Flower stage	7.15	7.73	8.11	7.79	8.95	7.82	9.37	7.83	9.51
W14	Tiller stage	3.23	3.53	9.29	3.61	11.76	3.63	12.38	3.64	12.69
	Jointing stage	5.87	6.41	9.20	6.51	10.90	6.54	11.41	6.55	11.58
	Flower stage	6.79	7.32	7.81	7.43	9.43	7.48	10.16	7.50	10.46

Where MLAI and SLAI are the measured and simulated LAI, respectively. *N*_sp_ is the number of segmentations of each leaf (Fig 3). RE denotes the relative error.

## 4 Discussion

In previous studies [18, 21, 25, 49], the visualization simulations of the above-ground parts of rice plants were neither comprehensive nor meticulous. Most of these studies primarily focused on morphological modeling of the fully expanded leaves of rice plants, while neglecting the unexpanded blades. For this reason, we employed a spatial helical surface in conjunction with a leaf shape model [26] to simulate the dynamic morphology of unexpanded rice leaves, thereby improving the realism and accuracy of our simulations [27]. Meanwhile, Drawing upon our RGB models for leaf color [30] and panicle color [29], we can compute RGB values for various positions on the leaf or panicle. This approach enables us to capture the color variations over time and space. Furthermore, our models for the angle between stem and sheath [28], leaf curve [27], panicle curve [29], and panicle morphology [29] are utilized to enhance and refine the morphological modeling and 3D visualization of rice plant, The visualization effects are significantly more natural and realistic compared to previous studies [18, 21, 25] (Figs 4–7).

The above-ground part and root system constitute an integral, interconnected unit in rice plant. However, most previous efforts have primarily concentrated on the above-ground components, overlooking the root system [18, 21, 25]. Consequently, these were lack of integrity and systematism. Drawing upon our lab’s outcomes [14, 26–30, 50, 51], we have achieved a 3D dynamic visualization of the entire rice plant over GDD. This visualization considers the synchronized relationship between the above-ground part and root system, as depicted in Fig 10. This comprehensive approach provides valuable support for the study of rice plant phenotypes, enabling a deeper understanding of their morphological and physiological characteristics.

Given the variations among rice individuals, there is an increased demand for efficient algorithms and rendering techniques that can optimize both the rendering efficiency and realism of plant populations. In this study, the differences between rice individuals are made by randomizing organ morphological and structural parameters as well as dynamically randomly allocating tiller number based on the simulation with the model of tiller number [48]. Concurrently, we improve the visualizations of rice populations by incorporating multi-technology fusion algorithms that are founded on grid model simplification and LOD. These advancements significantly boost both the rendering efficiency and realism of the visualizations.

In this study, we employed a leaf segmentation method grounded in our virtual rice model to simulate the LAI of two rice cultivars across different growth stages. Comparisons between measured and simulated LAI reveal that the simulated LAIs tend to exceed measured values, but they maintain acceptable deviations by the evaluation with RE, RMSE, MAE, and R^2^ (Table 1 and Fig 13). This slight overestimation was mainly caused from considering yellow leaves in the computation of leaf area, whereas such leaves were not considered in the measurement of rice LAI. This discrepancy in simulated LAI could potentially result in deviations in crop growth status assessment and yield forecasts. As the number of leaf segmentations increases, the lost leaf area decreases, resulting a corresponding increase in LAI. However, our analysis revealed no significant differences in the errors associated with different segmentation levels (Table 1). If the quantity of leaf segmentations is insufficient, the virtual rice lacks realism. Conversely, an excessive amount would lead to a surge in computational demand. Therefore, a number of 10 for leaf segmentations is deemed appropriate. The virtual rice model can simulate the plant phenotype continuously, enabling predictions of crop growth status and production throughout the growing cycle, without the need for actual field experiments. This approach alleviates the constraints of research reliant on traditional crop field experiment [53, 54].

In addition to LAI, the virtual rice model also allows for the extraction of various rice phenotype parameters, including plant height, leaf angle, organ length, and others, which can then be compared with measured values for analysis. Rice morphology is complex and influenced by numerous factors, including nitrogen, water availability, planting density, pests, and diseases. To address these, future research will focus on enhancing and optimizing the morphological modeling and visualization capabilities of the virtual rice model to adapt to different growth conditions, based on field experiments under different treatments. The improved models will then be able to predict a wider range of rice phenotype parameters, thereby providing valuable support for rice production and management.

## 5 Conclusion

Drawing upon the previous findings and the intricate synchronization relationships within rice plant, we have achieved 3D dynamic simulations of the above-ground components, root system, rice individuals, and rice populations, leveraging computer programming and graphic technique. Furthermore, our virtual rice model has demonstrated excellent results in simulating the LAI for two rice cultivars across various growth stages, where RE spans from 7.58% to 12.69%, as well as the RMSE, MAE, and R^2^ are 0.56, 0.55, and 0.86, respectively. These advancements offer valuable models and technical support for 3D visualizations of other crop plants, as well as their application in crop production and management.

## Supporting information

S1 Data (XLSX)
